# Wen-Luo-Tong Prevents Glial Activation and Nociceptive Sensitization in a Rat Model of Oxaliplatin-Induced Neuropathic Pain

**DOI:** 10.1155/2016/3629489

**Published:** 2016-08-25

**Authors:** Bo Deng, Liqun Jia, Lin Pan, Aiping Song, Yuanyuan Wang, Huangying Tan, Qing Xiang, Lili Yu, Dandan Ke

**Affiliations:** ^1^Department of Oncology of Integrative Chinese and Western Medicine, China-Japan Friendship Hospital, Beijing 100029, China; ^2^School of Clinical Chinese Medicine, Beijing University of Chinese Medicine, Beijing 100029, China

## Abstract

One of the main dose-limiting complications of the chemotherapeutic agent oxaliplatin (OXL) is painful neuropathy. Glial activation and nociceptive sensitization may be responsible for the mechanism of neuropathic pain. The Traditional Chinese Medicine (TCM) Wen-luo-tong (WLT) has been widely used in China to treat chemotherapy induced neuropathic pain. However, there is no study on the effects of WLT on spinal glial activation induced by OXL. In this study, a rat model of OXL-induced chronic neuropathic pain was established and WLT was administrated. Pain behavioral tests and morphometric examination of dorsal root ganglia (DRG) were conducted. Glial fibrillary acidic protein (GFAP) immunostaining was performed, glial activation was evaluated, and the excitatory neurotransmitter substance P (SP) and glial-derived proinflammatory cytokine tumor necrosis factor-*α* (TNF-*α*) were analyzed. WLT treatment alleviated OXL-induced mechanical allodynia and mechanical hyperalgesia. Changes in the somatic, nuclear, and nucleolar areas of neurons in DRG were prevented. In the spinal dorsal horn, hypertrophy and activation of GFAP-positive astrocytes were averted, and the level of GFAP mRNA decreased significantly. Additionally, TNF-*α* mRNA and protein levels decreased. Collectively, these results indicate that WLT reversed both glial activation in the spinal dorsal horn and nociceptive sensitization during OXL-induced chronic neuropathic pain in rats.

## 1. Background or Introduction

Oxaliplatin (OXL) is a third-generation chemotherapeutic agent commonly used to treat metastatic colorectal cancer [1]. However, one of its main dose-limiting complications is painful neuropathy [2]. The primarily sensory symptoms occur in up to 60% of patients, and they commonly have a delayed manifestation that can linger for many years [3, 4]. The signs of neuropathy start with paresthesia, followed by hyperesthesia [5] and a heightened sensitivity to cold/low temperatures [6]. Thus, these patients suffer from both the physical symptoms and a decreased quality-of-life [1]. According to the American Society of Clinical Oncology no agents are currently recommended to prevent/treat these abnormal sensations [6]. And the presently available pain drugs only offer marginal relief due to either a lack of efficacy or the risk of unacceptable side effects [1].

The superficial dorsal horn of the spinal cord is the first synaptic site for pain transmission from peripheral afferent nerves to the central nervous system. Dysfunction in neuroglial communication within the spinal dorsal horn plays a key role in neuropathic pain induced by OXL [7, 8]. This dysfunction arises from the activation of glia and increased production of glial-derived proinflammatory cytokine (tumor necrosis factor-*α*, TNF-*α*), resulting in modified neurotransmission [9]. The modified release of excitatory neurotransmitter (substance P, SP) also contributes to OXL-induced neuropathic pain [10]. This dysfunction increases peripheral nerve injury-induced central sensitization in the spinal cord [7–10].

There is no study on the effects of Traditional Chinese Medicine (TCM) on glial activation in the spinal dorsal horn induced by chronic OXL administration. In this study, we examined the effects of herbal medicine Wen-luo-tong (WLT) on glial activation in the spinal dorsal horn in a rat model of OXL-induced neuropathic chronic pain. We also examined the expression of excitatory neurotransmitter SP and glial-derived proinflammatory cytokine TNF-*α* in the spinal dorsal horn.

## 2. Materials and Methods

### 2.1. Preparation of Wen-Luo-Tong

The constituents of WLT are shown in Table 1. All TCM herbs, purchased from the Pharmacy of China-Japan Friendship Hospital, were identified and authenticated by the Head of the Department. The voucher specimens are available in our laboratory. According to the Chinese Pharmacopoeia 2010, extract amounts of component herbs were weighed according to the classic percentage and mixed well. The mixture was soaked in 10 volumes of distilled water (v/w) for 1 h. The mixture was boiled for 40 min and extracted twice. This preparatory method followed the ancient method [11] and was also identical to clinical preparatory method. The supernatant was concentrated to 0.6 g/mL in a water bath. The concentration of WLT was expressed as the total dry weight of the crude herb per milliliter in decoction.

### 2.2. Animals

All animal procedures were performed according to the Guidelines of Beijing (China) for the Ethical Use of Animals (2010). A total of 60 specific pathogen-free (SPF) female Wistar rats weighting 180–200 g were purchased from Beijing HFK Bioscience Co., LTD (certification number SCXK (Jing) 2007-0001). They were housed at the Animal Department of Clinical Institute in China-Japan Friendship Hospital (occupancy permit: SYXK (Jing) 2005-0019). Animals were acclimatized for 1 week before experiments. They were then randomly divided into 3 groups (20 in each group) according to body weight: the control group (normal control), the model group (blank control), and the WLT group. Of the 20 rats in each group, 8 were used for behavioral tests and ELISA, 6 for histological studies, and 6 for RNA extraction.

### 2.3. Drugs and Treatments

OXL (Sanofi–Aventis, China) was dissolved in 5% glucose at 1 mg/mL and stored at 4°C. As described previously [12], OXL (4 mg/mL) was intraperitoneally (i.p.) injected twice weekly with a cumulative dose of 36 mg/kg, in the model group and the WLT group. Rats from the control group were injected with 5% glucose (vehicle) according to a similar schedule. As described previously [13, 14], rats from the WLT group were placed in special cells (dimensions, 30 × 10 × 10 cm). Their paws and tails were soaked in WLT (25–28°C, 20 min, twice daily) from days 1 to 30.

### 2.4. Nociceptive Behavioral Tests

The presence of mechanical allodynia and hyperalgesia was assessed using calibrated von Frey hairs (4 and 15 g), which were applied to the plantar surface of the hind paw with increasing force until the individual filament started to bend [15]. The filament was applied for 1-2 s, and the procedure was repeated five times at 4-5 s intervals. Paw withdrawal responses (%) were calculated by taking ten (five per paw) repeated stimuli (4 or 15 g). Paw movement associated with locomotion or weight shifting was not counted as a withdrawal response.

### 2.5. Pathological Analysis of Dorsal Root Ganglia

On day 31, animals were sacrificed under pentobarbital sodium anesthesia. The whole blood was withdrawn from each animal by cardiac puncture using a heparin-coated syringe. Dorsal root ganglia (DRG) were rapidly dissected as described previously [16]. Muscle and connective tissue were partly removed from the spinal column in search of white nerves. Lumbar 4–6 (L4–6) dorsal root nerves were slightly pulled from the intervertebral foramen using curved ophthalmic forceps, and the bulge was identified as ganglion. DRG were fixed in 4% paraformaldehyde and embedded in paraffin. For morphometric analysis, sections were stained with haematoxylin and eosin (HE). Morphometric determinations of the somatic, nuclear, and nucleolar areas of neurons in DRG were performed using Image-Pro Plus (IPP) software (Media Cybernetics, USA).

### 2.6. Immunohistochemistry Analysis of Spinal Cord

Sections of the L4–6 spinal cord (paraformaldehyde fixed and paraffin embedded) were processed for immunohistochemistry using a primary antibody against glial fibrillary acidic protein (GFAP) (1 : 600, Wanleibio, China). Following incubation overnight at 4°C, sections were incubated in a horseradish peroxidase-conjugated secondary antibody for 4 h at room temperature. The color was developed with 3,3′-diaminobenzidine (DAB). The integral optical density (IOD) and area of hypertrophy in GFAP-positive cells were evaluated using IPP software.

### 2.7. RNA Extraction, Reverse Transcription, and Real-Time Polymerase Chain Reaction

Total RNA of the L4–6 spinal cord was extracted using TRIzol reagent (Invitrogen, USA). The reverse transcription reaction for first-strand cDNA synthesis was performed using reverse transcriptase (ABI, USA) with 2 *μ*g of total RNA. Quantitative real-time polymerase chain reaction (PCR) analysis was performed with the ABI 7500 real-time PCR system (ABI, USA). Real-time PCR primer sequences were listed in Table 2. The thermocycling reaction consisted of an initial denaturation at 95°C for 10 min, followed by 40 cycles at 95.0°C for 15 s and 60°C for 60 s. To confirm the specificity of amplification, a melting curve analysis was added, determining dissociation of amplified products from 60°C to 95°C. The mean value of the replicates for each sample was calculated and expressed as the threshold cycle (CT). The fold change in GFAP level or TNF-*α* level normalized by GAPDH level was determined by 2^−ΔΔCT^ method.

### 2.8. Enzyme-Linked Immunosorbent Assay

Five mL blood samples collected by cardiac puncture were centrifuged at 3000 rpm/min for 15 min at 4°C. The plasma was collected in 1.5 mL sterile Eppendorf tubes and stored at −20°C for enzyme-linked immunosorbent assay (ELISA). Concentrations of SP in plasma and L4–6 spinal cord homogenate, and concentrations of TNF-*α* in L4–6 spinal cord/DRG homogenate were measured using a double antibody sandwich ELISA kit (SUNBIO, China) according to the manufacturer's instructions. The concentrations of SP and TNF-*α* were individually calculated using specific standard curves.

### 2.9. Statistical Analysis

Data were analyzed with International Business Machines Corporation (IBM) Statistical Product and Service Solutions (SPSS) Statistics software 19.0 (IBM SPSS, USA). ANOVA followed by least significant difference (LSD) test was used. Ranked data (behavioral analysis) and measurement data (biochemical analysis) with non-Gaussian distribution or unequal variance were analyzed by the nonparametric Kruskal-Wallis test. A *P* value of less than 0.05 was considered significant.

## 3. Results

### 3.1. Mechanical Allodynia (Figure 1, Table 3)

Compared to the control group, paw withdrawal responses (%) of rats in the model group increased significantly (*P* < 0.01) for von Frey filament 4 g, indicating that OXL treatment caused mechanical allodynia. Additionally, paw withdrawal responses (%) of rats in the TCM group decreased significantly (*P* < 0.05) compared to rats in the model group.

### 3.2. Mechanical Hyperalgesia (Figure 1, Table 3)

In rats of the model group, increased responses (%) (*P* < 0.01) were observed for von Frey filament 15 g. OXL-induced mechanical hyperalgesia was relieved by TCM treatment, and responses (%) decreased significantly (*P* < 0.01).

### 3.3. Morphometric Examination of Dorsal Root Ganglion (Figure 2, Table 4)

Morphometric examination revealed that there was a significant difference in the somatic, nuclear, and nucleolar areas of neurons in DRG in the model group after treatment. The decrease in these areas induced by OXL was marked compared to that in the controls (*P* < 0.01). In TCM group, somatic, nuclear, and nucleolar areas were significantly larger than those of the model group (*P* < 0.01 or *P* < 0.05).

### 3.4. Glial Fibrillary Acidic Protein Staining in the Spinal Cord (Figure 3, Table 5)

**Figure 2:**
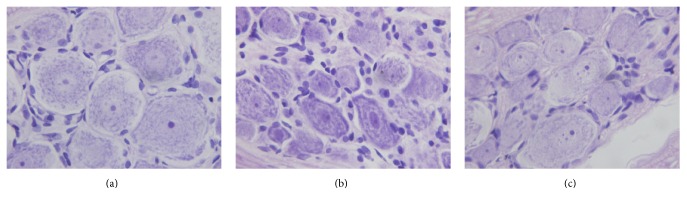
Pathological analysis of dorsal root ganglia (DRG) (×1000). (a) Control group: no morphometric change. (b) Model group: decreases in somatic, nuclear, and nucleolar areas induced by OXL. (c) TCM group: somatic, nuclear, and nucleolar areas were significantly larger as compared to model group.

Immunohistochemistry revealed a marked increase in GFAP reactivity in astrocytes from rats of the model group, with enlargement of astrocyte soma and increased astrocytic processes (astrocytosis) perivascularly and within the glia limitans (Figure 2). The IOD and GFAP-positive area within astrocytes increased significantly in comparison to that in the controls (*P* < 0.01). In TCM group, these pathological changes were alleviated significantly as compared to the model group (*P* < 0.01).

### 3.5. Changes in Protein Levels of Substance *P* and Tumor Necrosis Factor-Alpha (Table 6)

In rats of the model group, OXL treatment significantly increased SP and TNF-*α* levels in the spinal cord compared to levels in the controls (*P* < 0.01). In rats of the TCM group, TNF-*α* level in the spinal cord decreased significantly compared to the level in the model group (*P* < 0.01). The TNF-*α* level in DRG and SP levels also decreased in rats of the TCM group; however, the difference was not statistically significant.

### 3.6. Analysis of Glial Fibrillary Acidic Protein and Tumor Necrosis Factor-Alpha mRNA by Quantitative Real-Time Polymerase Chain Reaction (Figure 4, Table 7)

In OXL-treated rats, the mRNA expression of GFAP and TNF-*α* in the spinal cord increased significantly (*P* < 0.01) compared to the mRNA levels in the controls. WLT treatment significantly decreased GFAP and TNF-*α* mRNA levels (*P* < 0.05 or *P* < 0.01) compared to the levels in the model group.

## 4. Discussion

Chinese herbal medicine has been administered locally for thousands of years in China. TCM transdermal penetration enhancers could increase local drug concentration and alleviate local pain. Systemic administration could reduce local drug concentration and at the same time cause adverse reactions and complications. In this study, the effects of TCM on glial activation and nociceptive sensitization were studied for the first time in a rat model of OXL-induced chronic neuropathic pain.

In this rat model of OXL-induced chronic neuropathic pain, mechanical allodynia and mechanical hyperalgesia were observed (*P* < 0.01). Morphometric changes in neurons of DRG, including a reduction in nuclear, somatic, and nucleolar areas (*P* < 0.01), were consistent with previous observations [8, 12]. Hypertrophy of astrocytes and an increase in GFAP immunohistochemical reactivity (*P* < 0.01) were also observed. The activation of GFAP-positive astrocytes is associated with the development of mechanical allodynia and hyperalgesia. In addition, increased SP and TNF-*α* mRNA expression was observed in spinal cord. These results are consistent with those reported previously [7–10], suggesting that spinal glial activation and increased production of proinflammatory cytokines and excitatory neurotransmitters might contribute to the maintenance of OXL-induced chronic neuropathic pain in rats.

WLT has been used in clinical practice for nearly 20 years. A randomized, multicenter, double-blinded, controlled clinical trial involving 102 patients illustrated that WLT treatment significantly relieved chemotherapy induced peripheral neuropathy [11]. This study demonstrated that WLT suppressed the spinal activation of astrocytes following OXL administration and alleviated OXL-induced mechanical allodynia and mechanical hyperalgesia (*P* < 0.05 or *P* < 0.01). In spinal dorsal horn, hypertrophy and activation of GFAP-positive astrocytes were prevented (*P* < 0.01), and the level of GFAP mRNA decreased significantly (*P* < 0.05). In addition to their supportive role in neuronal health, glial cells strongly influence neuronal signaling due to their ability of regulating the uptake or release of neuroactive substances, including proinflammatory cytokines. Once released, proinflammatory cytokines, such as TNF-*α*, not only can act on neurons resulting in enhanced excitability, but can also give feedback on glial cells leading to an amplification loop that could potentially be responsible for the long-lasting hypersensitivity [8, 17]. WLT prevented the increasing of TNF-*α* mRNA and protein levels in the spinal cord, thereby disrupting the amplification loop. Many investigators contend that the accumulation of OXL or its metabolite, oxalate, in DRG is attributed to the high sensitivity and excitability of the peripheral nerve [12, 18]. This study demonstrated that WLT also alleviated the reduction in the somatic, nuclear, and nucleolar areas (*P* < 0.01 or *P* < 0.05) of neurons in DRG induced by OXL.

WLT is comprised of four herbs and their chemical constituents are complex. Epimedium extract effectively promotes peripheral nerve regeneration and improves the function of damaged nerves in a rat model of sciatic nerve crush [19]. Icariin, the main ingredient of Epimedium extract, is neuronal protective, acting as a nerve growth factor-releasing agent. Local application of icariin after spinal injury can promote peripheral nerve regeneration [20, 21].* Geranium wilfordii* and* Carthamus tinctorius* are used in TCM as analgesic anti-inflammatory agents. Geranium wilfordii extracts have inhibitory effects on the primary inflammatory factor, TNF-*α* [22]. Additionally,* Carthamus tinctorius* extracts possess remarkable antinociceptive and anti-inflammatory activities in mice models of inflammatory pain [23]. Cassia Twig, which is mostly used in Huangqi Guizhi Wuwu Decoction, is effective in the treatment of CIPN or diabetic peripheral neuropathy in clinical practice [24, 25]. Cinnamaldehyde from Cassia Twig can be absorbed by human skin and it is also a transdermal enhancer [26].

However, it is still unclear which constituent(s) is responsible for the effects of WLT in OXL-induced neuropathic pain. The molecular targets and mechanisms of these constituents require further investigation. In our ongoing studies, we are employing network pharmacology methods to identify the constituents of WLT and its molecular targets.

## 5. Conclusions

In a well-controlled OXL-induced chronic neuropathic pain model, we showed for the first time that WLT reversed both spinal glial activation and nociceptive sensitization.

## Figures and Tables

**Figure 1:**
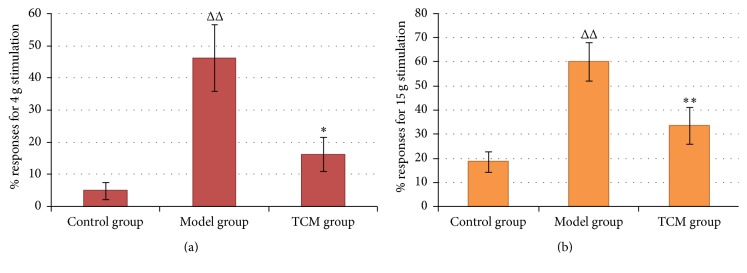
Percentages of paw withdrawal responses to mechanical stimulation. (a) Mechanical allodynia: paw withdrawal responses (%) to mechanical stimulation by von Frey filament 4 g. (b) Mechanical hyperalgesia: paw withdrawal responses (%) to mechanical stimulation by von Frey filament 15 g. Kruskal-Wallis test, ^ΔΔ^ *P* < 0.01, compared with control group; ^*∗*^ *P* < 0.05 and ^*∗∗*^ *P* < 0.01, compared with model group.

**Figure 3:**
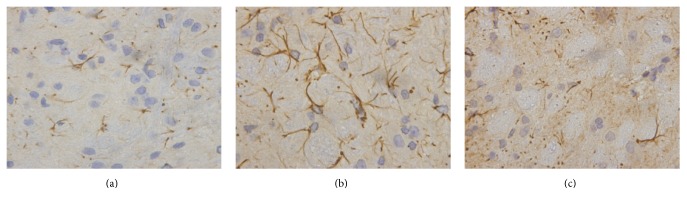
GFAP stain in spinal cord (×1000). (a) Control group: no astrocytic hyperactivation. (b) Model group: enhanced immunolabeling for GFAP within the superficial dorsal horn. (c) TCM group: spinal astrocytic hyperactivation was prevented.

**Figure 4:**
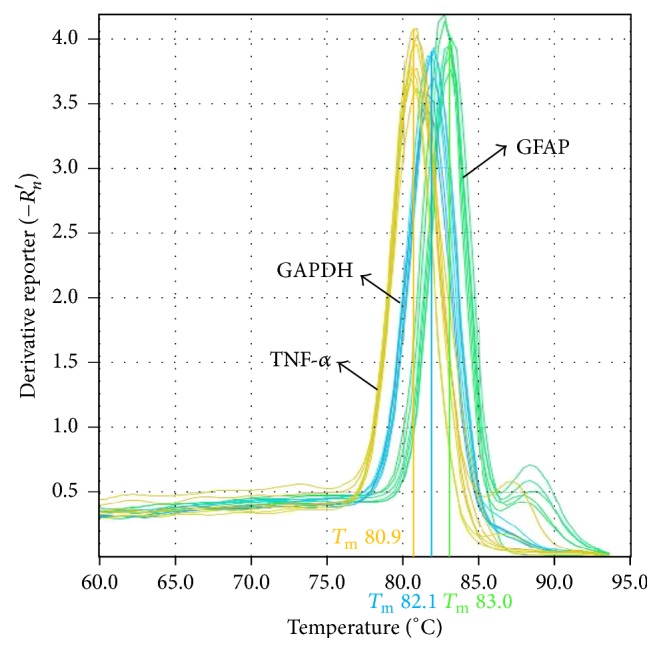
Melt curve analysis of GFAP, TNF-*α*, and GAPDH. It demonstrates the single product-specific melting temperatures: GFAP (80.9°C), TNF-*α* (83.0°C), and GAPDH (82.1°C). No primer-dimer formation was produced during 40 amplification cycles.

**Table 1:** Constituents of Wen-luo-tong.

Botanical name	Chinese name	English name	Weight (g)	Voucher numbers
*Epimedium brevicornum Maxim. *	Yinyanghuo	Epimedium herb	30	1508113
*Geranium wilfordii Maxim. *	Laoguancao	Geranium wilfordii	20	302415101
*Cinnamomum cassia Presl.*	Guizhi	Cassia Twig	15	SA7181
*Carthamus tinctorius L.*	Honghua	Carthamus tinctorius	10	SA8221

**Table 2:** Primer sequence for real-time PCR.

Gene	Primer sequence	
GFAP	Forward	5′-CCAAGATGAAACCAACCT-3′
	Reverse	5′-CGCTGTGAGGTCTGGCTT-3′

TNF-*α*	Forward	5′-GGCCACCACGCTCTTCTGTC-3′
	Reverse	5′-GGGCTACGGGCTTGTCACTC-3′

GAPDH	Forward	5′-GCCAAGGCTGTGGGCAAGGT-3′
	Reverse	5′-TCTCCAGGCGGCACGTCAGA-3′

**Table 3:** Percentages of paw withdrawal responses to mechanical stimulation (*n* = 8).

Group	Mechanical allodynia (%)	Mechanical hyperalgesia (%)
Control group	5.00 ± 5.35	18.75 ± 8.35
Model group	46.25 ± 20.65^ΔΔ^	60.00 ± 16.04^ΔΔ^
TCM group	16.25 ± 10.61^*∗*^	33.75 ± 15.06^*∗∗*^

Notes: Kruskal-Wallis test,  ^ΔΔ^ *P* < 0.01, compared with control group; ^*∗*^ *P* < 0.05 and ^*∗∗*^ *P* < 0.01, compared with model group.

**Table 4:** Morphometric analysis of DRG in different groups (*n* = 6).

Group	Somatic area (*μ*m^2^)	Nuclear area (*μ*m^2^)	Nucleolar area (*μ*m^2^)
Control group	2331.98 ± 420.66	332.27 ± 54.11	20.69 ± 4.74
Model group	335.67 ± 93.43^ΔΔ^	65.80 ± 13.11^ΔΔ^	3.81 ± 1.22^ΔΔ^
TCM group	1595.95 ± 304.03^ΔΔ*∗∗*^	275.43 ± 133.10^*∗∗*^	14.75 ± 2.99^*∗*^

Notes: one-way ANOVA or Kruskal-Wallis test,  ^ΔΔ^ *P* < 0.01, compared with control group; ^*∗*^ *P* < 0.05 and ^*∗∗*^ *P* < 0.01, compared with model group.

**Table 5:** Comparison of GFAP protein expression in spinal cord (*n* = 6).

Group	IOD	Area (*μ*m^2^)
Control group	0.55 ± 0.07	191.44 ± 171.04
Model group	1.27 ± 0.33^ΔΔ^	1366.17 ± 486.86^ΔΔ^
TCM group	0.61 ± 0.11^*∗∗*^	129.85 ± 54.31^*∗∗*^

Notes: one-way ANOVA,  ^ΔΔ^ *P* < 0.01, compared with control group;  ^*∗∗*^ *P* < 0.01, compared with model group.

**Table 6:** Comparison of SP and TNF-*α* protein levels in different groups (*n* = 8).

Group	SP (pg/mL)		TNF-*α* (pg/mL)	
	Spinal cord	Plasma	Spinal cord	DRG
Control group	51.43 ± 13.47	8.23 ± 4.62	119.23 ± 21.17	102.18 ± 17.52
Model group	156.83 ± 14.88^ΔΔ^	24.32 ± 9.32^ΔΔ^	329.14 ± 46.48^ΔΔ^	135.30 ± 37.05^Δ^
TCM group	110.43 ± 41.57^Δ^	17.42 ± 6.88^Δ^	226.48 ± 43.50^ΔΔ*∗∗*^	105.14 ± 0.82

Notes: one-way ANOVA or Kruskal-Wallis test, ^Δ^ *P* < 0.05 and ^ΔΔ^ *P* < 0.01, compared with control group;  ^*∗∗*^ *P* < 0.01, compared with model group.

**Table 7:** Comparison of GFAP and TNF-*α* mRNA expression in different groups (*n* = 6).

Group	GFAP (2^−ΔΔCT^)	TNF-*α* (2^−ΔΔCT^)
Control group	1.01 ± 0.16	1.02 ± 0.23
Model group	1.88 ± 0.57^ΔΔ^	2.21 ± 0.72^ΔΔ^
TCM group	0.96 ± 0.64^*∗*^	1.07 ± 0.40^*∗∗*^

Notes: Kruskal-Wallis test, ^ΔΔ^ *P* < 0.01, compared with control group; ^*∗*^ *P* < 0.05 and ^*∗∗*^ *P* < 0.01, compared with model group.
